# Overexpression of key complement regulators in glioblastoma

**DOI:** 10.1371/journal.pone.0349101

**Published:** 2026-05-15

**Authors:** Linnea Blomberg, Leonora Raba, Johan Bengzon, Kurt Osther, Henrietta Nittby Redebrandt

**Affiliations:** 1 Division of Clinical Sciences, Department of Neurosurgery, Rausing Laboratory, Lund University, Lund, Sweden; 2 Department of Neurosurgery, Skåne University Hospital, Lund, Sweden; Cedars-Sinai Medical Center, UNITED STATES OF AMERICA

## Abstract

**Background:**

Glioblastoma (GBM) is the most prevalent and malignant primary brain tumor in adults. While immune evasion is a well-recognized driver of GBM progression and a major obstacle for efficient immunotherapy, the role of the complement system remains underexplored. C1-inhibitor (C1-INH), a regulator of complement activation, was recently found overexpressed in GBM. We therefore hypothesized that GBM overexpresses additional complement regulators beyond C1-INH and the present work aimed to identify these.

**Method:**

Gene expression of complement inhibitors, the complement regulator pentraxin-3 (PTX3), and complement proteins was analyzed across nine publicly available transcriptomic datasets. Within each dataset, statistical comparisons were performed between sample groups for each gene. Differentially expressed complement inhibitors were validated at the protein level by immunostaining in the rat GBM cell line NS1 and patient derived GBM tissue.

**Results:**

*CFI*, encoding factor I, was significantly overexpressed in GBM compared to non-tumoral brain, while *THBD* and *CFH*, encoding thrombomodulin and factor H, displayed moderate overexpression. *SERPING1*, encoding C1-INH, was also upregulated, confirming previous findings. Immunostaining confirmed the expression of these inhibitors *in vitro* as well as in human glioblastoma tissue. Additionally, PTX3 and early complement proteins were significantly overexpressed in GBM, while levels of C5 and downstream components were comparable to normal brain.

**Conclusions:**

Our findings indicate that the GBM tumor overexpresses a specific set of complement regulators and components of the complement cascade, possibly inhibiting an efficient anti-tumoral immune response. Further investigations of these regulators as potential therapeutical targets in GBM are therefore highly warranted.

## Introduction

Glioblastoma (GBM) accounts for most primary malignant brain tumors in adults, with a global incidence of 3–4 cases per 100.000 [1]. Despite intensive research and multifaceted treatment approaches, GBM remains fatal with a median survival of approximately one year [2] and a 5-year survival rate of 7% [2,3].

A defining challenge of GBM is its capacity for immune evasion [4]. The tumors are immunologically quiet, presenting with few neoantigens, while upregulating the expression of immune checkpoints and immunosuppressive cytokines, such as transforming growth factor-β and interleukin-10. This prevents the immune activation, and instead promotes recruitment of suppressive immune cells, including myeloid-derived suppressor cells and regulatory T cells (T-regs) [4]. Whereas immunotherapies have emerged as highly efficient treatments against several hard-to-treat cancers, the profound and complex immunosuppression hinders this effect in GBM [5]. Given that immunotherapeutic success requires a functional immune system, various strategies to overcome GBM-induced immunosuppression have been proposed. These include cytokine therapies to promote pro-inflammatory signaling, enhancing the proliferation and activity of T cells, and preventing the recruitment and immunosuppressive polarization of tumor-associated macrophages, or alternatively, increasing their phagocytic activity [4,5].

**Fig 1:**
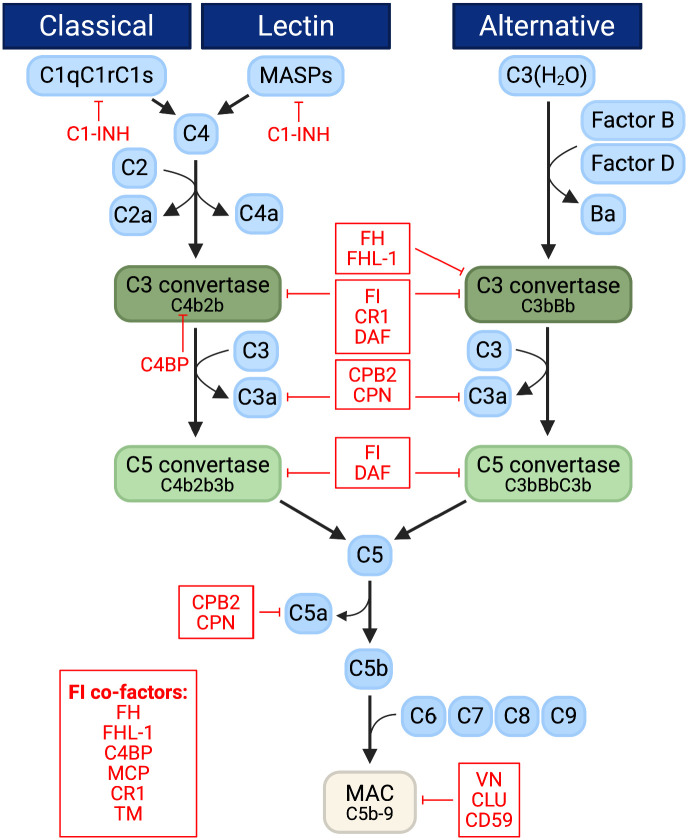
Overview of the complement system. The complement system includes three main pathways: the classical, lectin and alternative, which all converge at the activation of C5. Released anaphylatoxins (C3a and C5a) have immunological functions, whereas the membrane attack complex (MAC) inserts into and disrupts the membrane of the target. Complement inhibitors listed as FI co-factors promote regulatory activity of FI. MASP = mannose-binding lectin-associated serine protease; C1-INH = C1-inhibitor; C4BP = C4 binding protein; FH = factor H; FHL-1 = factor H-like protein 1; FI = factor I; CR1 = complement receptor 1; DAF = decay-accelerating factor; CPB2 = carboxypeptidase B2; CPN = carboxypeptidase N; MCP = membrane cofactor protein; TM = thrombomodulin; VN = vitronectin; CLU = clusterin; CD59 = protectin..

While immune cells of the GBM microenvironment have already been thoroughly investigated, the role of the complement system remains elusive, despite being a major component of the innate immune system [6]. The complement system comprises a series of proteins which are sequentially cleaved and activated, serving to opsonize and destroy pathogens and abnormal cells while bridging innate to adaptive immunity. C3a and C5a, known as anaphylatoxins, are released throughout this cascade, promoting inflammation through chemotaxis, degranulation, and immune cell activation [7]. There are three main pathways of the complement system: the classical, the lectin and the alternative (Fig 1) [6]. Although their triggers and initial stages differ, they all converge at the activation of complement protein C5 and proceed through a common cascade, culminating in the formation of the membrane attack complex (MAC). The MAC penetrates the cell membrane of pathogens and abnormal cells to form a pore, eventually causing them to burst [8]. Human cells are partially protected from MAC insertion by the MAC inhibitor protectin (CD59), which, when expressed on certain cancer cells, promotes tumor growth [9]. Although systemic complement proteins are primarily produced by the liver, tumor cells can also produce them locally [6], indicating that tumors actively modulate complement activity in their microenvironment. The mechanisms of activation, predominant pathways, and extent of complement response induced by cancer, however, remain poorly understood and likely vary significantly across cancer types [10]. While exerting anti-tumoral effects in some cancers, consistent with its classical role of protection, the complement system promotes tumor progression in others by driving immunosuppression, angiogenesis and proliferation [6]. In particular, anaphylatoxins have been implicated in shaping an immunosuppressive tumor microenvironment by recruiting suppressive immune cell populations and impairing anti-tumor T cell responses [11]. In GBM, complement activation has also been linked to the maintenance of stem-like cell niches, promoting tumor proliferation [12]. Accordingly, multiple therapeutic strategies have focused on inhibiting anaphylatoxins [11] or modulating complement signaling pathways through upstream regulators [13]. However, the functional outcome of complement activation is highly context-dependent and is thought, at least in part, to be determined by its extent. Whereas low-levels may cause chronic inflammation, promoting tumor initiation and growth, strong activation can mount a robust anti-tumoral response, potentially sufficient to eliminate cancer cells [14].

The complement system, being a potent and dynamic cascade, is tightly regulated to avoid overactivation [15]. Tumor cells can exploit this by increasing the expression of complement regulatory proteins, contributing to immune evasion [12,16]. One such regulator, C1-inhibitor (C1-INH), encoded by the *SERPING1* gene, dissociates the initial protein complexes of the classical and lectin pathways, thereby preventing their activation. Interestingly, C1-INH has been found to be overexpressed in GBM, suggesting its involvement in tumor-mediated immunosuppression [17]. Supporting this, rats intracranially inoculated with GBM cells pre-treated with anti-C1-INH exhibited a survival benefit compared to those inoculated with treatment-naïve cells. Further investigation revealed that intratumoral injections of anti-C1-INH also prolonged survival in rats with subcutaneous GBM, both as a monotherapy [18] and combined with irradiation [19]. However, when administered to rats with intracranial GBM, no survival benefit was observed [19]. Although this may have resulted from insufficient dosing or the partly intact blood-brain barrier (BBB) limiting efficacy, it could also indicate that the complement system must be targeted at multiple sites to elicit an immunological response within the brain and tumor microenvironment. While anti-C1-INH treatment in combination with hypofractionated radiotherapy increased the levels of early complement mediators, including C1r, C1s, C2, C3, and C4b, downstream proteins remained unaffected, suggesting the presence of additional complement inhibitors, which would also have to be targeted in order to enhance complement activation [19]. Indeed, GBM cells have been found to actively produce other complement inhibitors, including CD59, factor H (FH), and factor H-like protein 1 (FHL-1) [20]. Several studies have shown that blocking the regulatory function of these, including CD59 [21] and FH [22], reduces tumor growth, suggesting their target potential in cancer treatment.

Complement regulation is not mediated solely by complement inhibitors, in fact, several other proteins are involved, either directly or indirectly. Pentraxins are a family of pattern recognition molecules, comprising both short (C-reactive protein and serum amyloid protein P) and long proteins (neuronal pentraxin 1, neuronal pentraxin 2, neuronal pentraxin receptor, pentraxin 3 [PTX3], and pentraxin 4), which have a dual relationship with the complement system [23,24]. On one hand, these promote activation of the classical pathway by binding C1q and, in some cases, stimulate the lectin pathway by interacting with other activating components, thereby enhancing immune protection [23]. On the other hand, pentraxins engage with complement inhibitors such as C4 binding protein (C4BP) and FH, modulating their activity and suppressing the cascade at the C3b stage to prevent tissue damage. Thus, pentraxins are recognized as both inflammatory and anti-inflammatory mediators. PTX3 has recently emerged as a protein of interest in GBM, as it is overexpressed and associated with impaired survival [25,26].

The dual effects of complement have positioned the system as a potential therapeutic target in GBM, with emerging strategies aimed at either inhibiting complement activity to limit tumor-promoting inflammation or enhancing complement activation to boost anti-tumoral immunity [11]. Importantly, these seemingly opposing approaches are not inherently contradictory, but may instead reflect distinct strategies tailored to specific tumors and different mechanisms of immune regulation. Together, they underscore the need to better understand the balance between complement activation and regulation in cancer. The incomplete understanding of the complement system in GBM, specifically, represents a critical research gap, which may hold valuable insights for the development of more effective treatments against this devastating disease. This project aimed to investigate the gene expression of complement regulators and mediators in GBM compared to non-tumoral brain, with a particular focus on complement inhibitors, based on the hypothesis that these are differentially expressed in GBM.

## Materials and methods

### Gene expression data acquisition

Fourteen complement inhibitors (Table 1) were selected for gene expression analysis based on their well-established roles as regulators of the complement system [15,20,27,28]. C1-INH was included to validate previously reported overexpression [17]. Additionally, genes encoding major complement proteins (C1q, C1r, C1s, C2, C3, C4, C5, C6, C7, C8, and C9) and the complement regulator PTX3 were analyzed to provide broader context. Nine datasets (Table 2) containing transcriptomic high-throughput screening or microarray data of human GBM and control samples were retrieved from the Gene Expression Omnibus (GEO) DataSets database, provided by the National Center for Biotechnology Information (NCBI). They were identified using the search term “glioblastoma” and filtered for “expression profiling” to exclude non-transcriptomic data, yielding 1,732 datasets. To ensure reliable analyses, only datasets with ≥ 40 total samples were considered, leaving 311. These were manually reviewed to include patient-derived GBM samples and to exclude those lacking non-tumoral brain tissue controls, containing pre-treated samples, or consisting of pediatric cases, resulting in 39 datasets. From these, the 10 datasets with the largest number of GBM samples were selected to maximize statistical robustness and representativeness. One dataset was subsequently removed because the control samples consisted of stem cells, which have a distinct transcriptomic profile that could bias comparisons, leaving a final set of 9 datasets for analysis. The data were manually quality-checked for inconsistencies, such as extensive incomplete data or abnormal expression patterns. While most datasets included only GBM tumors and non-tumoral brain, three also contained a third sample group. Pairwise comparisons of gene expression were performed between all groups within the same dataset, resulting in two to three analyses per dataset.

**Table 1:** Complement inhibitors analyzed in this study, their encoding genes, and roles in complement regulation.

Complement inhibitor	Gene	Function
C1-inhibitor (C1-INH)	*SERPING1*	Binds and inactivates C1r, C1s, MASP-1 and MASP-2 [15]
C4 binding protein (C4BP)	*C4BPA C4BPB*	Binds C4b to prevent C3 convertase (C4bC2b) formation; has decay accelerating activity on C3 convertase (C4bC2b); FI co-factor [15,27]
Factor H (FH) Factor H-like protein 1 (FHL-1)	*CFH*	Have decay accelerating activity on C3 convertase (C3bBb); inactivate C3b; FI co-factors [15,20]
Factor I (FI)	*CFI*	Cleaves and inactivates C3b and C4b in presence of its co-factors FH, FHL-1, C4PB, MCP, CR1, TM [15]
Membrane cofactor protein (MCP)	*CD46*	FI co-factor [15]
Complement receptor 1 (CR1)	*CR1*	Has decay accelerating activity on C3 convertases; FI co-factor [15]
Decay-accelerating factor (DAF)	*CD55*	Has decay accelerating activity on C3 and C5 convertases [15]
Vitronectin (VN)	*VTN*	Binds C5b-7 to prevent MAC insertion, forming SC5b-9; inhibits C9 polymerization [15]
Clusterin (CLU)	*CLU*	Binds C5b-7 to prevent MAC insertion, forming SC5b-9; inhibits C8 and C9 polymerization [15]
Protectin (CD59)	*CD59*	Prevents C9 binding to C5b-8, inhibiting MAC formation [15]
Carboxypeptidase B2 (CPB2)	*CPB2*	Inactivates C3a and C5a [28]
Carboxypeptidase N (CPN)	*CPN1*	Inactivates C3a and C5a [28]
Thrombomodulin (TM)	*THBD*	Enhances co-factor activity of FH; co-factor of thrombin-mediated CPB2 activation [15]

MASP = mannose-binding lectin-associated serine protease; MAC = membrane attack complex.

**Table 2:** Overview of analyzed datasets, including tissue types, pairwise comparisons, Gene Expression Omnibus (GEO) accession numbers, data types, data generation methods, and sample counts.

ID	Comparison	Accession	Description	Samples
1 1.1 1.2 1.3	PGBM vs. NB RGBM vs. NB PGBM vs. RGBM	GSE263588	2024; RNAseq via HTS; patient-derived cell lines and NB samples obtained at time of diagnosis and recurrence [29]	PGBM n = 41 RGBM n = 4 NB n = 4
2	GBM vs. NB	GSE196533	2022; RNAseq via HTS; 39 of GBM samples are primary and treatment-naïve, however it is not specified which ones; NB samples from NIH NeuroBioBank [30]	GBM n = 61 NB n = 9
3 3.1 3.2 3.3	GBM vs. NB LGG vs. NB GBM vs. LGG	GSE147352	2021; rRNA-depleted RNAseq via HTS; GBM and LGG status (primary, recurrent or treatment-naïve) not specified; NB samples from NIH NeuroBioBank [31]	GBM n = 85 LGG n = 18 NB n = 15
4	GBM vs. NB	GSE121720	2018; rRNA-depleted ssRNAseq data via HTS; GBM samples collected at time of primary diagnosis; post-mortem NB samples from frontal, occipital, parietal and temporal brain [32]	GBM n = 60 NB n = 4
5	GBM vs. NB	GSE108474	2018; microarray gene expression; 76 samples overlapping with GSE4290 removed to avoid reanalysis; GBM status (primary, recurrent or treatment-naïve) not specified [33]	GBM n = 164 NB n = 8
6	GBM vs. NB	GSE61335	2014; microarray gene expression; GBM status (primary, recurrent or treatment-naïve) not specified; NB obtained during epilepsy surgery or autopsy from cerebral cortex donors without neurological disorders [34]	GBM n = 48 NB n = 14
7	GBM vs. NB	GSE22866	2011; microarray gene expression; GBM samples collected from newly diagnosed patients, NB samples obtained during epilepsy surgery [35]	GBM n = 40 NB n = 6
8 8.1 8.2 8.3	PGBM vs. NB RGBM vs. NB PGBM vs. RGBM	GSE7696	2008; microarray gene expression [36]	PGBM n = 70 RGBM n = 10 NB n = 4
9	GBM vs. NB	GSE4290	2006; microarray gene expression; GBM status (primary, recurrent, or treatment-naïve) not specified; NB obtained during epilepsy surgery [37]	GBM n = 81 NB n = 23

IDs denote datasets and comparison groups, with the main number indicating the dataset and the sub-number indicating the pairwise comparison. PGBM = primary glioblastoma; RGBM = recurrent glioblastoma; NB = non-tumoral brain tissue; GBM = glioblastoma; LGG = low-grade glioma; HTS = high-throughput screening; NIH = National Institutes of Health.

### Cell culturing

Green fluorescent protein (GFP)-positive NS1 rat GBM cells [38], generated and provided by the Rausing laboratory at Lund University in 2015, were used for immunocytochemistry. The cell line was derived in accordance with ethical standards at the time of its creation, and has been extensively tested for tumor inoculation, demonstrating the growth of histopathologically verified IDH-wt GBM with necrosis and vascular dissemination. NS1 cells were cultured in Iscove’s Modified Dulbecco’s Medium (IMDM) (Gibco) supplemented with 0.5% gentamicin, 1% Na-pyruvate, 1% Gluta-MAX (Gibco) and 10% heat-inactivated fetal bovine serum (FBS), the latter of which had been heated to 56°C for 30 min prior to medium preparation. The cells were incubated at 37°C in a humidified 5% CO_2_ incubator.

Upon completion of culturing, the medium was removed, and the cells were washed once with phosphate-buffered saline (PBS). Trypsin (TrypLE™ Express Enzyme 1X, Gibco) was added for 5 min to detach the cells, after which 10X the volume of medium was added to inactivate the enzyme. The suspension was centrifuged at 500g for 5 min, the supernatant was removed, and the cell pellet was resuspended in a minimal volume of medium. The cell suspension was mixed 1:1 with 0.4% Trypan Blue Solution (Gibco) and loaded onto a hemocytometer for cell counting. Sterile glass coverslips were placed in a 24-well plate, and 50,000 cells per well were added in medium. The cells were cultured overnight at 37°C in a humidified 5% CO_2_ incubator.

### Immunostaining

#### Cell culture.

To confirm the expression of complement inhibitors identified as differentially expressed in GBM, immunocytochemistry was performed on NS1 cells. FH, factor I (FI), vitronectin (VN), and thrombomodulin (TM) were included as these were the complement inhibitors most significantly altered in our transcriptomic analysis, suggesting potential functional relevance in GBM. After approximately 2 days of culture, the medium was removed, and cells were washed three times with PBS, before being fixed in 4% paraformaldehyde (PFA) for 10 min. The cells were washed three more times, permeabilized using 0.025% Triton™ X-100 for 10 min, and blocked with PBS containing 1% bovine serum albumin (BSA) for 1 h.

Primary antibodies were diluted in PBS containing 1% BSA. The FI antibody (PA5–79036, Thermo Fisher Scientific), supplied lyophilized, was reconstituted in distilled water according to the instructions and then diluted 1:500. The TM antibody (PA5–120883, Thermo Fisher Scientific) was diluted 1:100, VN (15833–1-AP, Thermo Fisher Scientific) 1:50 and FH (MA5–50911, Thermo Fisher Scientific) 1:200. Cells were incubated overnight with primary antibodies at 4°C. Following incubation, the cells were washed three times with PBS, after which secondary antibodies, diluted 1:1000 in PBS containing 1% BSA, were added. Donkey anti-Rabbit Alexa Fluor™ Plus 594 (A32754, Invitrogen) was used for all targets except FH, for which Donkey anti-Mouse Alexa Fluor™ 594 (A-21203, Invitrogen) was used. The cells were incubated with secondary antibodies for 2 h at room temperature in darkness. Hoechst was diluted 1:1000 in PBS and added to the cells for 10 min, after which the cells were washed twice with PBS and once with MilliQ. The coverslips were mounted onto glass slides with mounting media, and the cells were imaged using a fluorescent microscope (Olympus VS120) with VS-ASW (S6) imaging software. Images were acquired at 20x magnification using automatic exposure settings, as the aim was to confirm expression rather than perform comparative analysis. Immunocytochemistry was performed using two technical replicates for each target. Negative controls were prepared using the same procedure, but omitting primary antibodies, to verify absence of strong autofluorescence and nonspecific labeling or binding. Negative controls verified the absence of strong red autofluorescence and nonspecific binding of secondary antibodies in NS1 cells (Materials and Methods Supplementary Data S3 Fig).

#### Human tumor tissue.

Human tumor tissue was collected prospectively after informed consent, in accordance with ethics permission from the Lund/Malmö Regional Ethical Review Board (permit number 2018/27). Three different types of tissue were used: normal tissue, tumor tissue, and mix tumor tissue/tumor front. To further validate the protein expression of complement inhibitors (FH, FI, VN, and TM) identified as differentially expressed in GBM, immunohistochemical staining was performed on paraffin-embedded human tissue sections using an avidin-biotin complex (ABC) method (VECTASTAIN® ABC Kit, Peroxidase Rabbit IgG).

Sections were deparaffinized in xylene and rehydrated through a graded ethanol series to distilled water. Antigen retrieval was performed using 1X citrate buffer (final pH 6) at 80°C for 20 min. Sections were blocked with a mixture of 3% hydrogen peroxide in 10% methanol in PBS for 10 min, followed by 5% normal goat serum for 30 min at room temperature. They were then incubated overnight at 4°C with primary antibodies diluted in 0.25% Triton™ X-100 in PBS (TPBS) containing 1% BSA. A total of four antibodies were used: FI 1: 200 (PA5–79036, Thermo Fisher Scientific), FH 1:200 (PA596080, Thermo Fisher Scientific), VN 1:100 (15833–1-AP, Thermo Fisher Scientific), and TM 1:200 (PA5–120883, Thermo Fisher Scientific).

Sections were then incubated with biotinylated secondary antibodies 1:200 for 1 h at room temperature, followed by incubation with the ABC peroxidase complex for 30 min. Between each step, sections were washed with 0.25% TPBS. Immunoreactivity was visualized using DAB as chromogen (BD Pharmingen™, DAB Substrate Kit). Sections were counterstained using Mayer´s hematoxylin and subsequently dehydrated through graded ethanols, cleared in xylene, and mounted with coverslips using xylene-based mounting medium. Negative controls verified the absence of strong auto-staining (Materials and Methods Supplementary Data S4 Fig)

### Statistical analysis

Data processing and statistical analyses were performed using R (version 4.4.2) and RStudio (version 2024.12.0 + 467). Gene expression data were log_2_-transformed for normalization and fold change calculation, unless stated that this had already been done or if the normalization method used by the dataset creators included log_2_ transformation. Welch’s t-test was used to calculate group mean differences and confidence intervals [39]. Multiple comparisons were corrected using the Bonferroni method. Gene expression differences with fold changes < 0.5 or > 2 and corrected p-values < 0.05 were considered significant.

## Results

### Differential expression of SERPING1, CFI, THBD, CFH and VTN in glioblastoma

**Fig 2:**
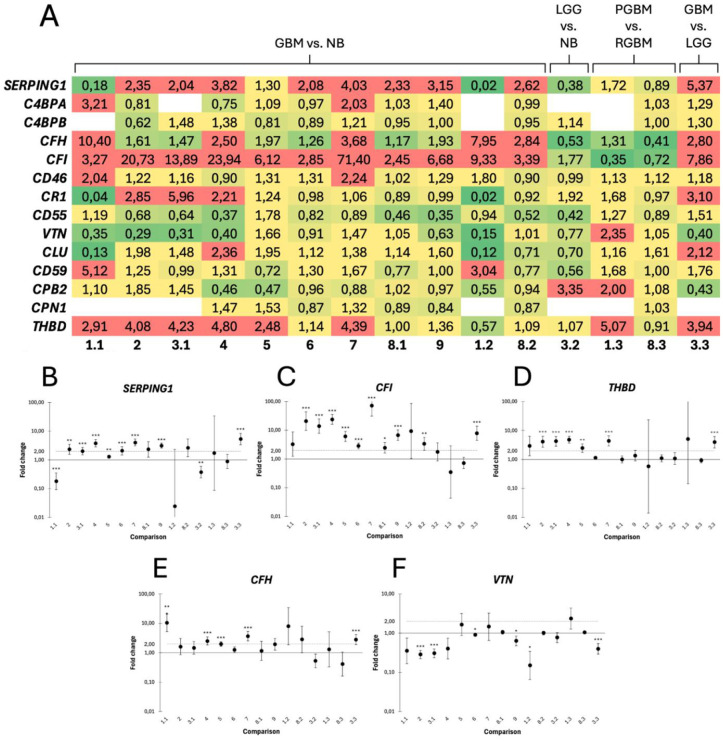
Differential expression of genes encoding complement inhibitors in glioblastoma (GBM) compared to non-tumoral brain. **A)** Heat map of fold changes for all analyzed complement inhibitors (rows) across each pairwise comparison (columns). Low fold changes are shown in green, transitioning through yellow to red, with red indicating fold changes ≥ 2. Empty cells indicate genes with insufficient measurable expression in one or more sample groups, preventing fold change calculation. **B-F)** Fold changes for complement inhibitors significantly altered in GBM. **B)** *SERPING1* and **C)** *CFI* were significantly overexpressed in most GBM versus control comparisons, **D)** *THBD* was upregulated in just under half of comparisons, **E)** *CFH* was modestly upregulated, and **F)** *VTN* was modestly downregulated. X-axis labels correspond to the comparisons as follows: 1.1 and 8.1, PGBM vs. NB; 1.2 and 8.2, RGBM vs. NB; 3.2, LGG vs. NB; 1.3 and 8.3, PGBM vs. RGBM; 3.3, GBM vs. LGG; all remaining columns represent GBM vs. NB. NB = non-tumoral brain tissue; PGBM = primary GBM; RGBM = recurrent GBM; LGG = low-grade glioma. Significance levels are indicated as *p < 0.05, **p < 0.01, and ***p < 0.001.

RNA sequencing data from human GBM and control samples were analyzed across 15 pairwise comparisons (Table 2) to assess the gene expression of complement inhibitors in GBM (Fig 2A). Consistent with previous reports, the analysis confirmed an overexpression of *SERPING1* (Fig 2B), encoding C1-INH, in GBM. *SERPING1* was upregulated in 9 of 11 comparisons between GBM and non-tumoral brain, with 8 presenting a fold change greater than 2 and 6 reaching statistical significance (p < 0.01). Comparing primary and recurrent GBM revealed no difference. Low-grade glioma (LGG) displayed a significant downregulation of *SERPING1* compared to non-tumoral brain, with expression levels less than one-fifth of that in GBM.

A prominent overexpression was observed for *CFI*, encoding FI. *CFI* was upregulated in all 11 comparisons exploring GBM versus non-tumoral brain, 9 of which were statistically significant (Fig 2C). Fold changes were above 10 in 4 of these, with p < 0.001. There was no significant difference in *CFI* expression between primary and recurrent GBM, despite the downregulation observed in both 1.3 and 8.3. *CFI* was significantly overexpressed in LGG compared to non-tumoral brain, although its expression was lower than that of GBM.

The analysis revealed upregulation of the TM-encoding gene *THBD* in GBM (Fig 2D). Of 11 comparisons between GBM and non-tumoral brain, 6 exhibited a fold change greater than 2, with 5 of those being statistically significant. There was no difference in *THBD* expression between primary and recurrent GBM. While *THBD* expression in LGG did not differ from that in non-tumoral brain, it was significantly downregulated compared to GBM.

*CFH*, encoding FH and FHL-1, was overexpressed in all 11 GBM versus non-tumoral brain comparisons (Fig 2E), with 5 exhibiting a fold change above 2 and 3 being significant (p < 0.001). Except for dataset 6, with a fold change just below 2, none of the other comparisons in this group reached statistical significance. There was no conclusive difference in *CFH* between primary and recurrent GBM. LGG had reduced *CFH* levels compared to non-tumoral brain and GBM, although the difference was only significant compared to GBM.

GBM exhibited a moderate downregulation of *VTN*, encoding VN (Fig 2F). Of the 11 GBM versus non-tumoral brain comparisons, 7 were downregulated. Five of these with values under 0.5, 3 of which reached statistical significance. *VTN* was upregulated in both datasets 1.3 and 8.3, comparing primary to recurrent GBM, however, neither were statistically significant. LGG exhibited an insignificant downregulation of *VTN* compared to non-tumoral brain, while GBM significantly downregulated *VTN* compared to LGG.

With the exception of *SERPING1* and *CD55*, no significant fold changes were found in dataset 3.2, which compared LGG to non-tumoral brain. Dataset 3.3, comparing GBM to LGG, showed upregulation of all measurable genes, except *VTN* and *CPB2*, with 6 genes reaching statistical significance. Fold changes comparing primary and recurrent GBM were largely inconclusive, with none of the genes being statistically significant. While dataset 1.3 showed a slight general overexpression of genes in primary GBM, dataset 8.3 exhibited fold changes close to 1. Fold changes of *CLU*, *CD46*, *CR1*, *CD55*, *CD59,* and *CPB2* were either inconclusive or appeared unchanged, while sufficient expression data for *C4BPA*, *C4BPB,* and *CPN1* were lacking in several datasets (S1 Fig).

### Factor I, thrombomodulin, factor H, and vitronectin expressed in NS1

**Fig 3:**
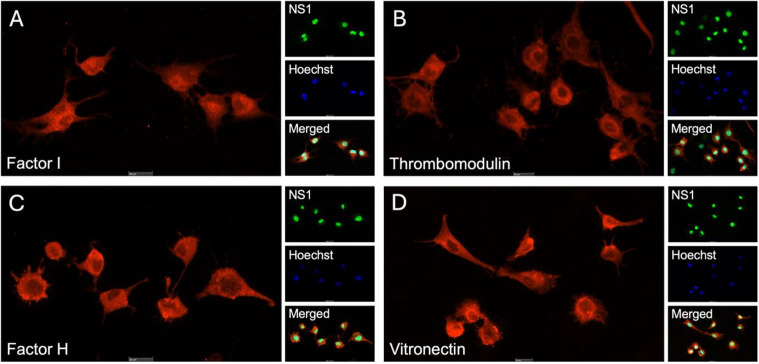
Immunocytochemical validation of complement inhibitor expression in NS1 cells. Complement inhibitors **A)** factor I, **B)** thrombomodulin, **C)** factor H and **D)** vitronectin are all expressed by NS1 at the protein level, as indicated with the red fluorescent (Alexa Fluor™ 594) signal. NS1 cells are an established green fluorescent protein-positive rat cell line used to model glioblastoma cells. Hoechst staining marks the cell nuclei (blue), while the final image of all four panels displays all fluorescent channels merged. Images include a scale bar indicating 20 μm.

To validate the expression of complement inhibitors FI, TM, FH and VN in GBM at the protein level, immunocytochemistry was performed on the GFP-positive NS1 rat GBM cell line. These proteins were selected as their corresponding genes were consistently and significantly altered in our transcriptomic analysis, indicating potential relevance to GBM pathology. AlexaFluor™ 594-conjugated secondary antibodies, emitting bright red signals in the fluorescent microscope, were used for detection. Immunocytochemistry detected fluorescent signals for all complement inhibitors in NS1 (Fig 3). The images demonstrate that AlexaFluor™ localizes to the cells, with the inhibitors appearing to be distributed either on the cell membrane or within the cytosol, but excluding the nuclei.

### Factor I, thrombomodulin, factor H, and vitronectin expressed in human glioblastoma versus non-tumoral brain tissue

**Fig 4:**
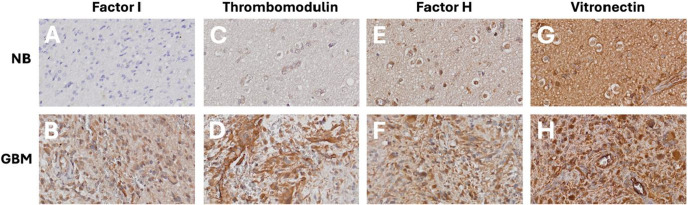
Immunohistochemical validation of complement inhibitor expression in non-tumoral brain (NB) and glioblastoma (GBM) tissue. **Representative images of A-B)** factor I (FI), **C-D)** thrombomodulin (TM), **E-F)** factor H (FH), and **G-H)** vitronectin (VN) are shown. NB tissue is displayed in the top row (A, C, E, G) and GBM tissue in the bottom row (B, D, F, **H)**. FI, TM, and FH were more strongly expressed in GBM than NB, whereas VN was strongly expressed in both tissues.

To assess the protein expression of FI, TM, FH, and VN in human GBM compared to non-tumoral brain tissue, immunohistochemistry was performed on tumor core tissue and brain tissue outside the tumor border, as defined by radiology (Fig 4). These proteins were selected based on their differential gene expression in GBM. FI, FH and TM were strongly expressed in tumor samples compared to non-tumoral brain tissue, consistent with the the transcriptomic analysis, which showed increased expression in GBM. Vitronectin, on the other hand, was expressed in both tumor and non-tumoral brain tissue.

### Pentraxin-3 markedly upregulated in glioblastoma

**Fig 5:**
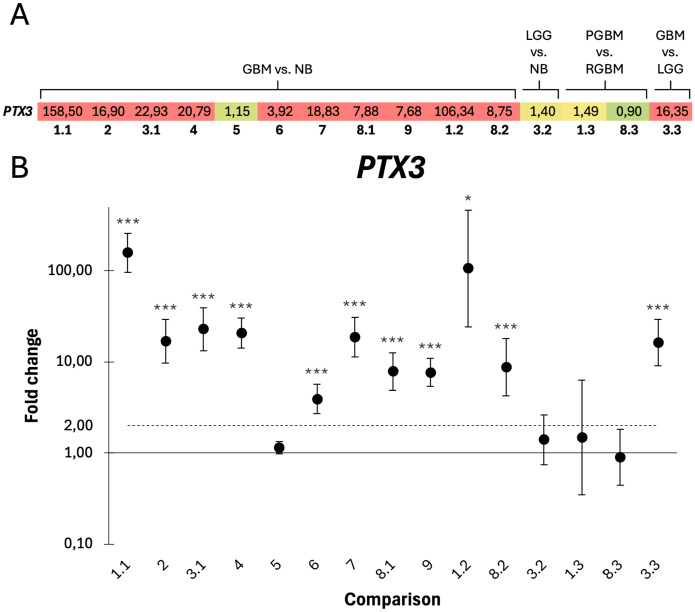
Differential expression of *PTX3* in glioblastoma (GBM) compared to non-tumoral brain. **A)** Heat map of *PTX3* fold changes across each pairwise comparison (columns). Low fold changes are shown in green, transitioning through yellow to red, with red indicating fold changes ≥ 2. **B)** *PTX3* was significantly overexpressed in all GBM versus non-tumoral brain comparisons, except for dataset 5. X-axis labels correspond to the comparisons as follows: 1.1 and 8.1, PGBM vs. NB; 1.2 and 8.2, RGBM vs. NB; 3.2, LGG vs. NB; 1.3 and 8.3, PGBM vs. RGBM; 3.3, GBM vs. LGG; all remaining columns represent GBM vs. NB. NB = non-tumoral brain tissue; PGBM = primary GBM; RGBM = recurrent GBM; LGG = low-grade glioma. Significance levels are indicated as *p < 0.05, **p < 0.01, and ***p < 0.001.

The expression of *PTX3* in GBM was assessed across 15 pairwise comparisons (Table 2, Fig 5A). *PTX3* was strongly upregulated in 10 of the 11 comparisons between GBM and non-tumoral brain, with 9 of these reaching statistical significance and 8 presenting p < 0.001 (Fig 5B). There were no significant differences observed in datasets 1.3 and 8.3, comparing primary and recurrent GBM. Similarly, dataset 3.2, which compares LGG to non-tumoral brain, exhibited an insignificant fold change. *PTX3*, however, was significantly upregulated in GBM compared to LGG (p < 0.001).

### Upregulation of early complement cascade proteins in glioblastoma

**Fig 6:**
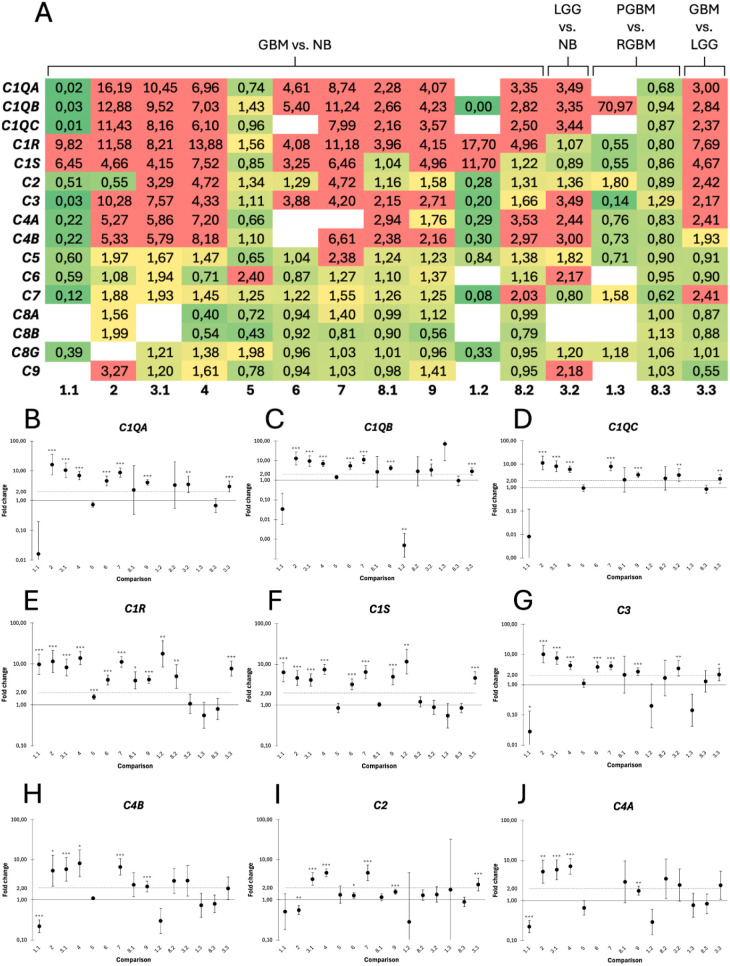
Differential expression of genes encoding complement proteins in glioblastoma (GBM) compared to non-tumoral brain. **A)** Heat map of fold changes for all analyzed complement proteins (rows) across each pairwise comparison (columns). Low fold changes are shown in green, transitioning through yellow to red, with red indicating fold changes ≥ 2. Empty cells indicate genes with insufficient measurable expression in one or more sample groups, preventing fold change calculation. **B-J)** Fold changes for complement proteins significantly altered in GBM. **B)** *C1QA*, **C)** *C1QB*, **D)** *C1QC*, **E)** *C1R*, **F)** *C1S*, **G)** *C3*, and **H)** *C4B* were consistently overexpressed across multiple datasets, whereas **I)** *C2* and **J)** *C4A* showed more variable upregulation, reaching statistical significance in only a subset of comparisons. X-axis labels correspond to the comparisons as follows: 1.1 and 8.1, PGBM vs. NB; 1.2 and 8.2, RGBM vs. NB; 3.2, LGG vs. NB; 1.3 and 8.3, PGBM vs. RGBM; 3.3, GBM vs. LGG; all remaining columns represent GBM vs. NB. NB = non-tumoral brain tissue; PGBM = primary GBM; RGBM = recurrent GBM; LGG = low-grade glioma. Significance levels are indicated as *p < 0.05, **p < 0.01, and ***p < 0.001.

To further explore the relationship between GBM and the complement system, the expression of 16 genes encoding major complement proteins or their subunits was investigated across 15 pairwise comparisons (Table 2). Complement mediators early in the cascade were frequently overexpressed (Fig 6A). *C1QA*, *C1QB*, *C1QC*, *C1R*, *C1S*, *C3* and *C4B* were significantly upregulated in GBM in at least 5 of the 11 comparisons between GBM and non-tumoral brain (Fig 6B-H). *C2* and *C4A* were upregulated in 8 and 6 of the 11 comparisons, respectively, but only 3 of these significantly (Fig 6I-J). Downstream mediators of the cascade, including *C5*, *C6*, *C7*, *C8G,* and *C9*, displayed fold changes close to 1, whereas expression data for *C8A* and *C8B* were absent throughout several datasets (S2 Fig).

Comparisons between primary and recurrent GBM revealed no pattern. Dataset 3.2, which compared LGG to non-tumoral brain, displayed an upregulation of all genes except for *C1R* and *C7*, while values for *C8A* and *C8B* were largely missing (Fig 6A). The upregulation was considered statistically significant for *C1QA*, *C1QB*, *C1QC* (Fig 6B-D), *C3* (Fig 6G) and *C5* (S2 Fig). Comparing GBM to LGG revealed a significant upregulation of *C1QA*, *C1QB*, *C1QC*, *C1R*, *C1S* (Fig 6B-F), *C2* (Fig 6I) and *C3* (Fig 6G) in GBM.

## Discussion

The present study investigated gene expression of complement proteins and regulators in GBM versus non-tumoral brain tissue across nine transcriptomic datasets. Our results confirmed the previously reported overexpression of *SERPING1* in GBM [17] and demonstrated a significant upregulation of *CFI*. Additionally, we revealed a moderate upregulation of *THBD* and *CFH*, and a downregulation of *VTN*. Immunostaining confirmed the expression of the corresponding inhibitors in NS1, a cell line modeling GBM, as well as in human GBM tissue compared to non-tumoral brain tissue. LGG was found to regulate complement inhibitors in the opposite direction, or to an attenuated degree compared to GBM. This finding aligns with previous research, highlighting the often opposite immunological mechanisms in LGG compared to high-grade glioma (HGG) [40]. Additionally, our study found a significant upregulation of *PTX3* in GBM compared to both non-tumoral brain and LGG, alongside a general upregulation of complement proteins operating early in the cascade, whereas downstream mediators did not exhibit this overexpression.

*CFI*, displaying a prominent upregulation in GBM, encodes the protease FI which inhibits the complement cascade by degrading C3b and C4b in the presence of its co-factors [15]. The expression of FI has previously been found correlated to a worse clinical outcome in breast cancer [41] and was recently proposed as a prognostic biomarker for glioma due to its local overexpression and correlation to survival [42]. FI knockdown was also demonstrated to effectively inhibit glioma cell proliferation, invasion and migration, *in vitro* and *in vivo*, while FI overexpression had the opposite effect. These findings suggest *CFI* as not only being an oncogene, but also a therapeutic target with the potential to impede tumor progression. FI inhibition would theoretically increase the release of anaphylatoxins, promoting a local anti-tumoral immune response, and could potentially decrease the activation of carcinogenesis-driving signaling pathways such as JAK/STAT and vascular endothelial growth factor, which have been found upregulated in FI^high^ glioma [42].

We observed a significant upregulation of *THBD*, encoding TM, in GBM. TM is an endothelial protein, regulating the complement system by promoting FI and CPB2 activation [15]. While mostly known for being an anticoagulant, it also has inflammation-controlling properties beyond complement regulation [43] and seems to have anti-tumoral effects. TM has been found to decrease cell proliferation, invasion and metastasis, and its expression is correlated to a favorable outcome in several cancers [44,45]. These observations are intriguing in the light of GBM, which has a particularly poor prognosis. The exact anti-tumoral mechanisms remain elusive, although thought to be at least in part independent of its anticoagulation activities [46], involving inhibition of the NF-κB pathway and epithelial-mesenchymal transition reversal [43]. *THBD* has been found overexpressed in gliomas [47,48], but inconsistent with our results, its expression was higher in LGG than HGG [49], which aligns with the understanding that TM offers a better prognosis. One possible reason for *THBD* being upregulated in GBM is that TM acts as a brake on thrombosis, frequently occurring with this disease.

Our study revealed a slight upregulation of *CFH* in GBM. *CFH* encodes complement inhibitors FH and FHL-1, regulating the cascade by preventing C3 convertase formation in the alternative pathway, accelerating decay of the C3 convertase and by being co-factors of FI [15,20]. These proteins have gained attention in recent years, and anti-tumoral effects beyond complement regulation have been identified. For instance, FH promotes tumor growth independent of the complement cascade [50], is correlated to tumor size and metastasis, and modulates the phenotype of macrophages in breast cancer, promoting an immunosuppressive environment [51]. FH has been found expressed by several cancers, is confirmed to help evade complement-mediated lysis [20] and is correlated to a more severe disease, earlier recurrence and a shorter survival [51–53]. FH has also recently been found to promote immunosuppression in glioma by increasing the viability and activity of T-regs [53]. These findings highlight FH as a potent tumor-promoting protein and suggest that its local inhibition is suitable to counteract GBM immunosuppression. Recent research identified a tumor-specific anti-FH autoantibody in non-small cell lung carcinoma, GT103, with compelling anti-tumoral activities *in vivo* [54]. While an anti-FH autoantibody is yet to be found for GBM, GT103 represents one promising approach through which FH can be targeted. FHL-1 is a truncated form of FH, produced by alternative splicing of *CFH*, retaining all functional domains and regulatory functions of FH [20]. Given limited data availability and challenges in obtaining suitable antibodies, FHL-1 expression was not assessed by immunocytochemistry.

VN is a functionally diverse protein in the extracellular matrix which drives tissue repair and metastasis by facilitating cell adhesion, matrix degradation and cell migration [55], while also maintaining vascular homeostasis, promoting angiogenesis [56], and preventing MAC formation [15]. Tumoral expression of VN is generally correlated to a poor survival [57] and, aligning with these observations, its levels have been found enhanced in HGG compared to both normal brain and LGG [58,59]. Contrastingly, we observed a downregulation of *VTN* in GBM. One possible explanation is that *VTN* expression varies within the tumor, resulting in differences depending on the region from which the samples are resected. VN has been found more intensely expressed in the surrounding stroma than in the tumor core [60] and specifically at the interface between glioma and adjacent brain [61]. The contradiction could also stem from VN being primarily produced in the liver and mainly reaching the tumor via vascular leakage [62]. Our deviating results may therefore reflect a lack of tumor-intrinsic VN production, as most of the protein might be supplied from external sources. Using immunocytochemistry and immunohistochemistry, we detected VN expression in the NS1 cell line as well as in GBM and non-tumoral brain tissue.

We observed a significant upregulation of *PTX3* in GBM, however, due to its dual relationship with the complement system [23], the exact effect of this overexpression is hard to determine without further assessing complement activation. While a high abundance of PTX3 may enhance both classical and lectin pathway activation through the binding of C1q and other mediators, it can also suppress the cascade. PTX3 binds FH and FHL-1, recruiting these inhibitors to sites of complement activity, thereby enabling their regulation of complement activation. Recent research outlines PTX3 as a key protein in GBM, both in terms of shaping a pro-tumoral immunosuppressive microenvironment and by promoting several carcinogenic processes. *PTX3* has been found overexpressed in GBM and is correlated with poor survival and increased immunosuppression through immune cell modulation [25]. The protein has also been associated with enhanced cell survival, angiogenesis, and invasion in GBM, suggesting PTX3 as a potential therapeutic target [63].

To investigate how GBM may regulate complement, we also examined the gene expression of of major complement proteins. When doing so, we observed a clear distinction between early and late mediators, as those operating before C5 were strongly upregulated, whereas a clear shift could be seen in the downstream proteins. Due to the complexity of the complement system and its dual role in tumor biology, it is possible that the GBM tumor may benefit from some degree of activation, particularly involving early complement mediators, whereas a full activation, including the terminal part of the cascade, is anti-tumoral.

There are certain limitations of the present study to consider. It should be noted that the gene expression does not necessarily reflect the presence or functional activity of the corresponding proteins. Immunostaining is a widely used and relevant follow-up technique to transcriptomic analysis for detecting protein expression, but it does not provide any precise information on its abundance. Although protein expression was assessed for selected complement inhibitors, quantitative validation was not performed. As an exploratory study with a primary focus on complement inhibitors, additional components of the cascade and PTX3 were included after the initial analysis to provide broader context. However, these were not further investigated at the protein level. Future studies should therefore incorporate quantitative protein analyses, functional assays, and validation of additional factors, such as PTX3, to better understand complement in GBM. For several genes (*C4BPA*, *C4BP*B, *CPN1*, *C8A* and *C8B)*, fold changes and p-values could not be calculated across multiple datasets, as one or both comparison groups either lacked gene expression data or contained only a single value. While technical issues in genetic profiling, data quality, or processing differences could explain the missing values, the repeated absence across datasets suggests that these genes are simply expressed at extremely low levels. Additionally, the limited number of non-tumoral brain tissue samples may reduce representativeness and biological variability. Furthermore, these samples were derived from diverse sources, including GBM and epilepsy patients, autopsies, and biobanks, which may not fully reflect normal brain physiology.

The strengths of this study lie primarily in its large set of data, uniquely covering 664 GBM samples in total. While our results may not fully capture the diversity of all GBM tumors due to high inter- and intratumoral heterogeneity, they provide a robust overview of how GBM may modulate complement activation. Importantly, the complement system exhibits dual and highly context-dependent roles in cancer. Understanding this balance is critical, as it may inform novel strategies aimed at shifting complement activity toward an anti-tumoral phenotype. Whereas previous studies have proposed inhibition of anaphylatoxins [11], our strategy instead focuses on restoring or enhancing complement activity. This may appear contradictory at first, but it reflects two distinct approaches to modulating the complement system, resulting in different levels of activation and potentially different outcomes [14]. Identifying the most suitable targets and an appropriate activation level may therefore be key to counteracting the immunosuppressive GBM microenvironment. Our findings suggest that targeting immune evasion through complement by inhibiting upregulated complement inhibitors could represent a novel avenue for immunotherapeutic intervention [68–71].

## Conclusions

In this study, we provide evidence for the upregulated expression of complement regulators FI, TM, FH and PTX3 in GBM through analysis of transcriptomic data from GBM tumors and non-tumoral brain. We further demonstrate that genes encoding early complement cascade proteins are overexpressed in GBM, whereas downstream mediators remain unaltered. These findings indicate that GBM selectively upregulates specific components of the complement system, underscoring their potential as therapeutic targets, particularly through inhibition of complement regulatory mechanisms.

## Supporting information

S1 Fig Complement inhibitors with unaltered, mostly non-significant, or insufficient gene expression data in glioblastoma (GBM) compared to non-tumoral brain. (DOCX)

S2 Fig Complement proteins with unaltered, mostly non-significant, or insufficient gene expression data in glioblastoma (GBM) compared to non-tumoral brain. (DOCX)

S3 Fig Negative control for immunohistochemistry in in NS1 cells. (DOCX)

S4 Fig Negative control for immunohistochemistry in human glioblastoma tissue. (DOCX)

## Abbreviations

ABC: avidin-biotin complex
BBB: blood brain barrier
BSA: bovine serum albumin
CD59: protectin
CLU: clusterin
CPB2: carboxypeptidase B2
CPN: carboxypeptidase N
CR1: complement receptor 1
C1-INH: C1-inhibitor
C4BP: C4 binding protein
DAF: decay-accelerating factor
FBS: fetal bovine serum
FH: factor H
FHL-1: factor H-like protein 1
FI: factor I
GBM: glioblastoma
GEO: Gene Expression Omnibus
GFP: green fluorescent protein
HGG: high-grade glioma
HTS: high-throughput screening
IDH: isocitrate dehydrogenase
IMDM: Iscove’s Modified Dulbecco’s Medium
LGG: low-grade glioma
MAC: membrane attack complex
MCP: membrane cofactor protein
NB: non-tumoral brain tissue
NCBI: National Center for Biotechnology Information
NIH: National Institutes of Health
PBS: phosphate-buffered saline
PGBM: primary glioblastoma
PFA: paraformaldehyde
PTX3: pentraxin-3
RGBM: recurrent glioblastoma
TM: thrombomodulin
TPBS: 0.25% Triton X-100 in PBS
T-regs: regulatory T cells
VN: vitronectin
