# Supplementary material for: Overexpression of key complement regulators in glioblastoma
Source: PLoS One. 2026 May 15;21(5):e0349101. doi: 10.1371/journal.pone.0349101 (PMC13178988; doi:10.1371/journal.pone.0349101)
Supplement: S1 Fig — (DOCX) [file pone.0349101.s001.docx]

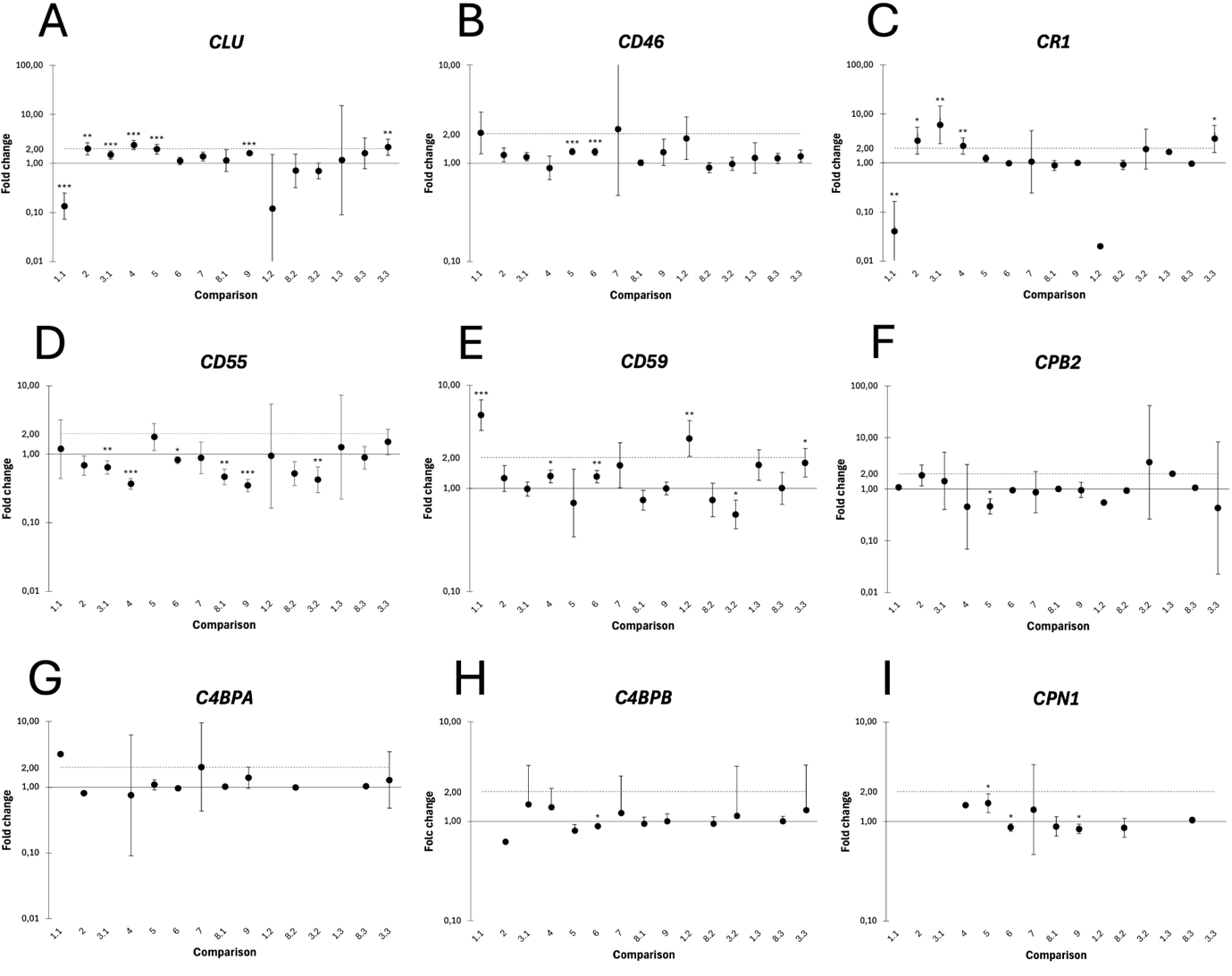


**Figure S1. Complement inhibitors with unaltered, mostly non-significant, or insufficient gene expression data in glioblastoma (GBM) compared to non-tumoral brain. A)** *CLU*, **B)** *CD46*, **C)** *CR1*, **D)** *CD55*, **E)** *CD59*, and **F)** *CPB2* showed largely unchanged or non-significant gene expression in GBM compared to non-tumoral brain. **G)** *C4BPA*, **H)** *C4BPB*, and **I)** *CPN1* lacked sufficient expression data across several datasets. X-axis labels correspond to the comparisons as follows: 1.1 and 8.1, PGBM vs. NB; 1.2 and 8.2, RGBM vs. NB; 3.2, LGG vs. NB; 1.3 and 8.3, PGBM vs. RGBM; 3.3, GBM vs. LGG; all remaining columns represent GBM vs. NB. NB = non-tumoral brain tissue; PGBM = primary GBM; RGBM = recurrent GBM; LGG = low-grade glioma. Significance levels are indicated as *p<0.05, **p<0.01, and ***p<0.001.
