# Supplementary material for: Overexpression of key complement regulators in glioblastoma
Source: PLoS One. 2026 May 15;21(5):e0349101. doi: 10.1371/journal.pone.0349101 (PMC13178988; doi:10.1371/journal.pone.0349101)
Supplement: S2 Fig — (DOCX) [file pone.0349101.s002.docx]

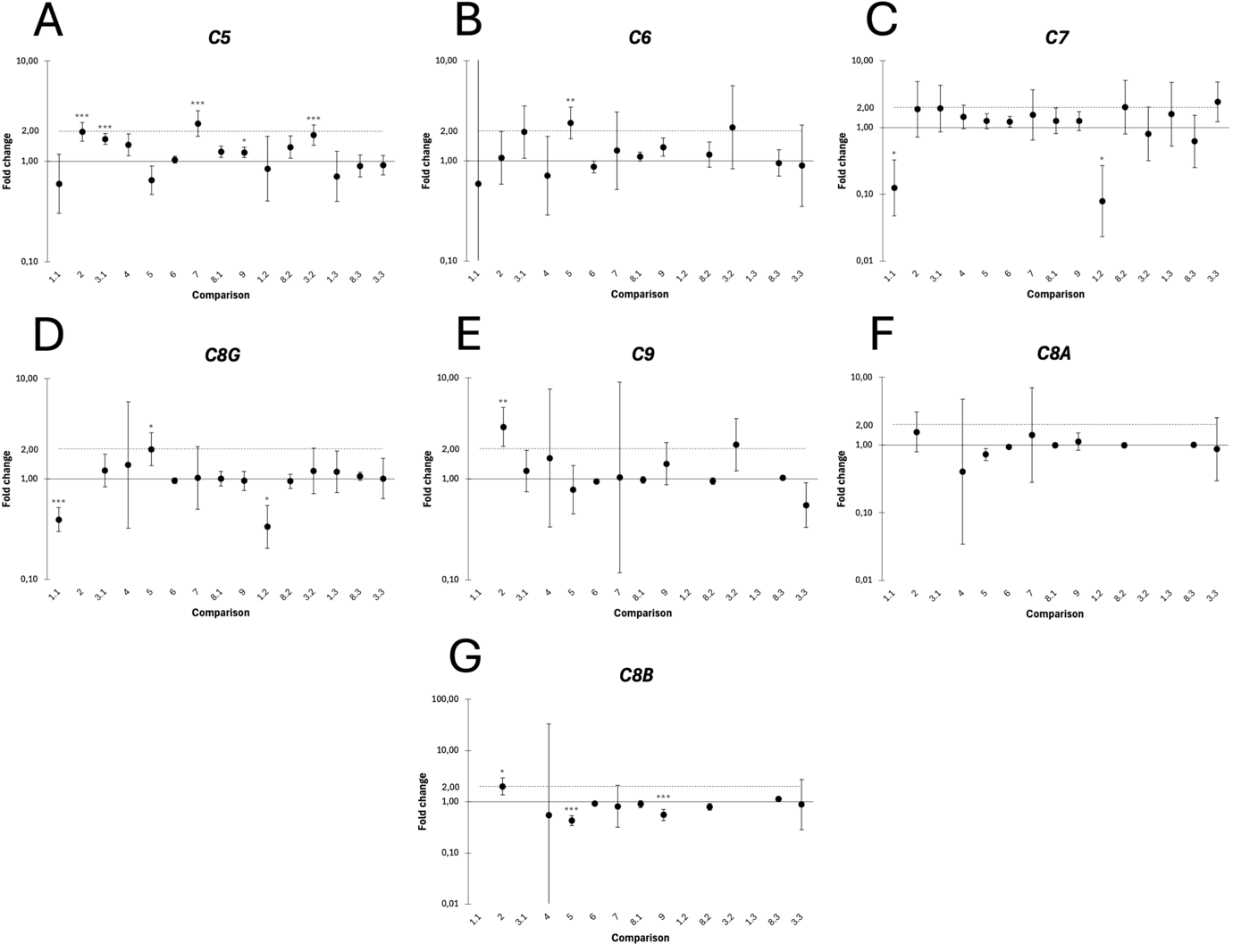


**Figure S2. Complement proteins with unaltered, mostly non-significant, or insufficient gene expression data in glioblastoma (GBM) compared to non-tumoral brain. A)** *C5*, **B)** *C6*, **C)** *C7*, **D)** *C8G*, and **E)** *C9* showed largely unchanged or non-significant gene expression in GBM compared to non-tumoral brain. **F)** *C8A* and **G)** *C8B* lacked sufficient expression data across several datasets. X-axis labels correspond to the comparisons as follows: 1.1 and 8.1, PGBM vs. NB; 1.2 and 8.2, RGBM vs. NB; 3.2, LGG vs. NB; 1.3 and 8.3, PGBM vs. RGBM; 3.3, GBM vs. LGG; all remaining columns represent GBM vs. NB. NB = non-tumoral brain tissue; PGBM = primary GBM; RGBM = recurrent GBM; LGG = low-grade glioma. Significance levels are indicated as *p<0.05, **p<0.01, and ***p<0.001.
