# Supplementary material for: Overexpression of key complement regulators in glioblastoma
Source: PLoS One. 2026 May 15;21(5):e0349101. doi: 10.1371/journal.pone.0349101 (PMC13178988; doi:10.1371/journal.pone.0349101)
Supplement: S3 Fig — (DOCX) [file pone.0349101.s003.docx]

**Materials and Methods Supplementary Data**


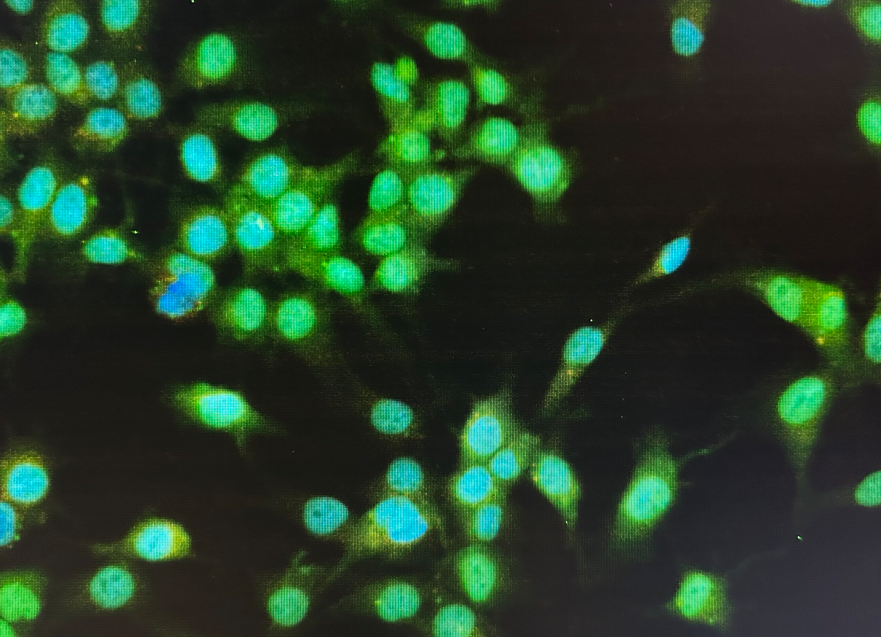


**Materials and Methods Supplementary Data Figure 1. Negative control for immunocytochemistry in NS1 cells.** NS1 cells were processed without primary antibodies to verify the absence of non-specific staining or autofluorescence. NS1 cells are an established green fluorescent protein-positive rat cell line used to model glioblastoma cells.
