# Supplementary material for: Overexpression of key complement regulators in glioblastoma
Source: PLoS One. 2026 May 15;21(5):e0349101. doi: 10.1371/journal.pone.0349101 (PMC13178988; doi:10.1371/journal.pone.0349101)
Supplement: S4 Fig — (DOCX) [file pone.0349101.s004.docx]

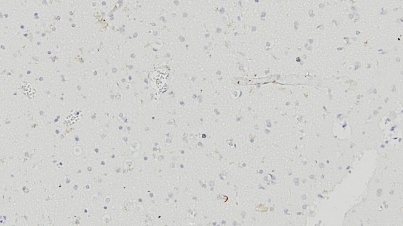


**Materials and Methods Supplementary Data Figure 2. Negative control for immunohistochemistry in human glioblastoma tissue.** A tissue section from outside the tumor core was processed without primary antibodies to verify the absence of non-specific staining.
